# Tris(2,2′-bi­pyridine)­iron(II) tris­(di­cyano­methylidene)methane­diide

**DOI:** 10.1107/S2414314625005036

**Published:** 2025-06-20

**Authors:** Said Abdelghafour Messalti, Fatima Setifi, Uwe Böhme, Zouaoui Setifi, Mohammad Hadi Al-Douh

**Affiliations:** a Laboratoire de Chimie Ingénierie Moléculaire et Nanostructures (LCIMN) Université Ferhat Abbas Sétif 1 Sétif 19000 Algeria; b Institut für Anorganische Chemie, Technische Universität Bergakademie Freiberg, Leipziger Str. 29, 09599 Freiberg, Germany; c Département de Technologie Faculté de Technologie Université 20 Août 1955-Skikda BP 26 Route d'El-Hadaiek Skikda 21000 Algeria; d Chemistry Department, Faculty of Science, Hadhramout University, Mukalla, Hadhramout, Yemen; Goethe-Universität Frankfurt, Germany

**Keywords:** crystal structure, solvothermal synthesis, iron(II) complex, 2,2′-bi­pyridine, tris­(di­cyano­methyl­idene)methane­diide

## Abstract

The asymmetric unit of the title compound, [Fe(C_10_H_8_N_2_)_3_][C{C(CN)_2_}_3_], contains an iron–bipyridyl unit and one third of two crystallographic independent tris­(di­cyano­methyl­idene)methane­diide units. As a result of crystallographic site symmetry the ratio of cations to anions is 1:1. The tris­(2,2′-bi­pyridine)­iron(II) cation has threefold symmetry. The two crystallographic independent tris­(di­cyano­methyl­idene)methane­diide ions are disordered over two atomic sites having equal occupancy. The anions have 3 symmetry. In the crystal, hydrogen bonds between cations and anions form complex layers parallel to (001). These are supplemented by hydrogen bonds perpendicular to the former, leading to a three-dimensional network.

## Abstract 2

The asymmetric unit of the title compound, [Fe(C_10_H_8_N_2_)_3_][C{C(CN)_2_}_3_], contains an iron–bipyridyl unit and one third of two crystallographic independent tris­(di­cyano­methyl­idene)methane­diide units. In the crystal, hydrogen bonds between cations and anions form complex layers parallel to (001). These are supplemented by hydrogen bonds perpendicular to the former, leading to a three-dimensional network.

## Graphical abstract

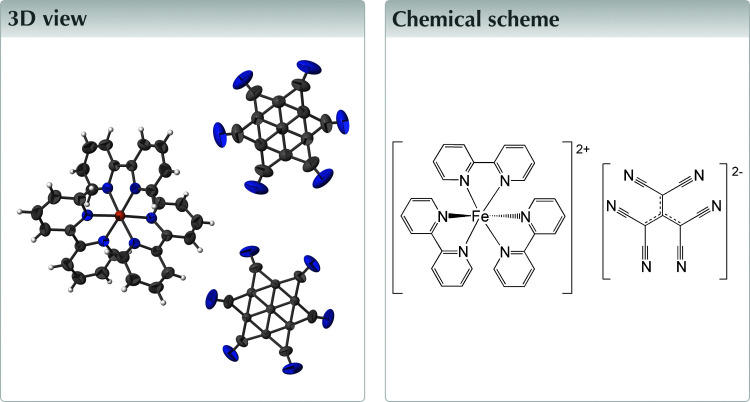

## Structure description

Organic cyano­carbanion anions have recently attracted considerable attention in the fields of coordination chemistry and mol­ecular materials (Benmansour *et al.*, 2010 ▸). As a consequence of their rigidity and electronic delocalization, these organic anions provide opportunities for the generation of mol­ecular architectures with varying dimensions and topologies (Benmansour *et al.*, 2008 ▸; Setifi *et al.*, 2010 ▸; Benamara *et al.*, 2021 ▸). Furthermore, the use of cyano­carbanion anions for the synthesis of inter­esting discrete and polymeric bis­table materials has been recently reported (Setifi, Milin *et al.*, 2014 ▸; Cuza *et al.*, 2021 ▸). It was during the course of attempts to prepare such materials with 2,2′-bi­pyridine as a co-ligand that the title complex was unexpectedly obtained. We report here the mol­ecular and supra­molecular structures of a new compound based on tris­(2,2′-bi­pyridine)­iron(II) and the tris­(di­cyano­methyl­idene)methane­diide dianion (tcpd^2−^) as the counter-ion.

**Figure 1:**
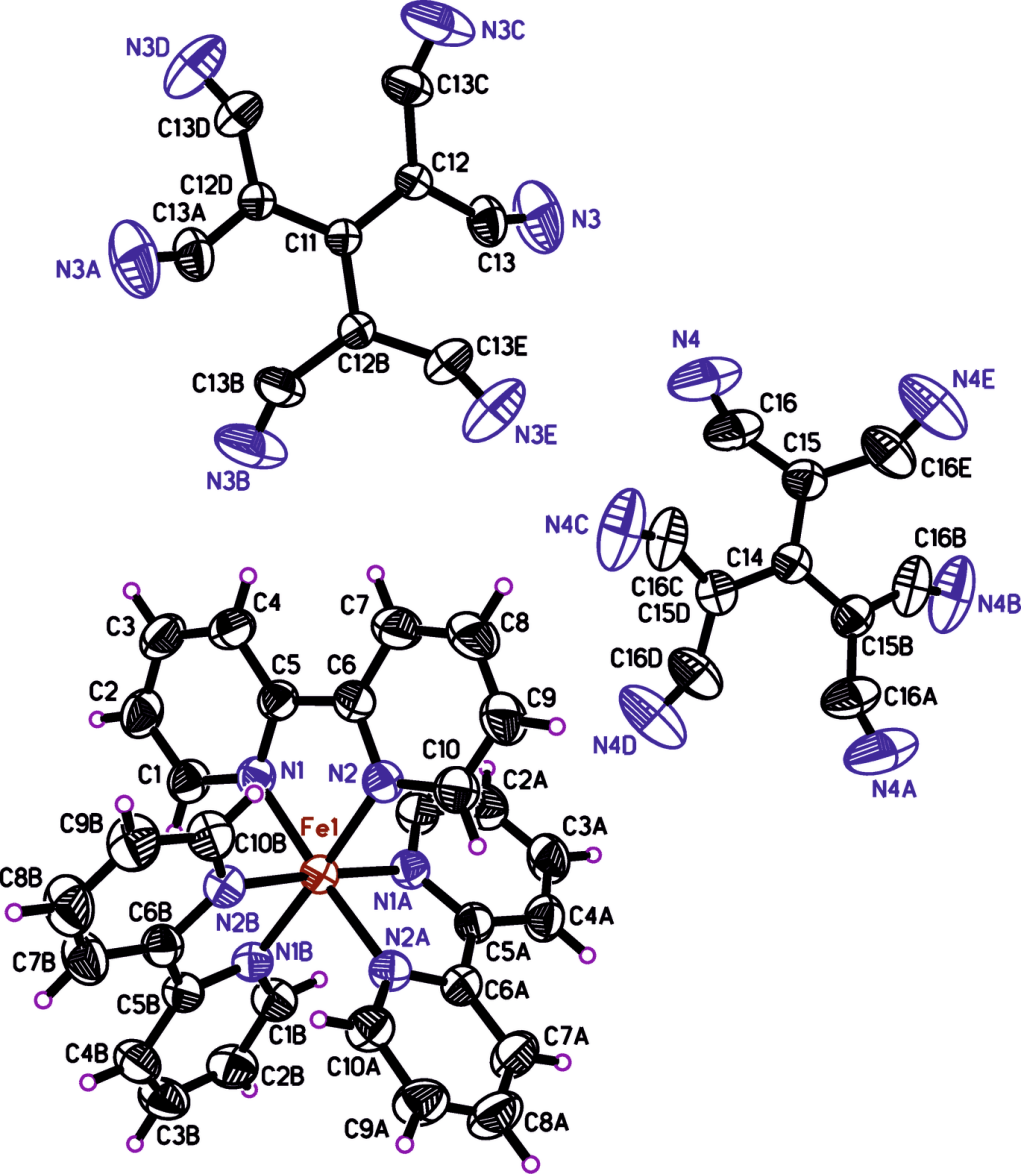
Mol­ecular structure of the title compound showing the atom-numbering scheme. Displacement ellipsoids are drawn with 50% probability displacement ellipsoids. Only one of the disordered set of sites is shown.

The crystal structure consists of an [Fe(C_10_H_8_N_2_)_3_]^2+^ cation with a six-coordinate iron atom in a slightly distorted octa­hedral coordination environment and a [C{C(CN)_2_}_3_]^2−^ anion (Fig. 1 ▸). At first glance, it is noticeable that two crystallographically independent anions are present. These have a site symmetry of 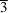, which means that one sixth is present in the asymmetric unit. The cation has site symmetry 3, *i.e.* it consists of an iron bipyridyl unit, with one third of the cation in the asymmetric unit. The resulting ratio of cation to anion is therefore 1:1. The two crystallographic independent tris­(di­cyano­methyl­idene)methane­diide ions are disordered over two atomic sites having equal occupancy, leading to a star-like appearance.

The Fe—N distances are comparable to other tris­(2,2′-bi­pyridine)­iron(II) complexes (Healy *et al.*, 1983 ▸; Setifi, Setifi *et al.*, 2014 ▸; Addala *et al.*, 2018 ▸). The angle N1—Fe1—N2 [81.40 (5)°] is determined by the bite angle of the bi­pyridine unit. The other *cis* angles in the coordination polyhedron deviate from 90° (see Table 1 ▸), as the octa­hedral cation is subject to compression in the direction of the threefold rotation axis.

**Figure 2:**
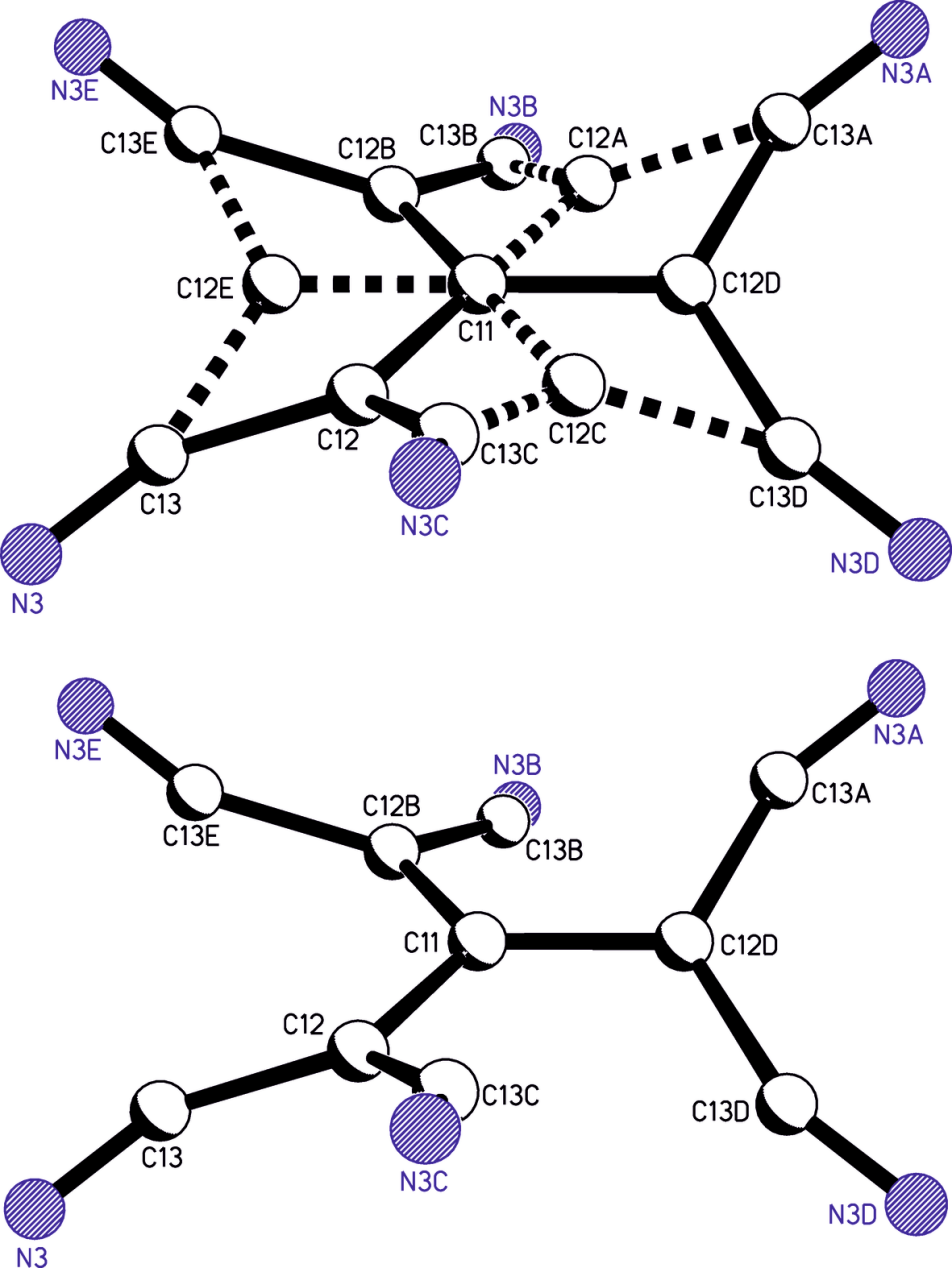
Side view of the anion C11—C12—C13—N3 with all symmetry-equivalent atoms (top). One of the two orientations is drawn with dashed bonds. Side view with one part of the disordered anion (bottom).

The tris­(di­cyano­methyl­idene)methane­diide dianions are disordered at the methyl­idene carbon atoms C12 and C15. The cyano end groups C13—N3 and C16—N4 show slightly elongated displacement ellipsoids due to the disorder at the neighbouring atoms. The cores of the anions (atoms C11/C12 and C14/C15 with their symmetry equivalents, respectively) are exactly planar. The cyano groups are twisted out of these planes (Fig. 2 ▸), making dihedral angles with it of 28.0 (2)° (N3, C13, C12, C13*C*, N3*C*) and 29.6 (2)° (N4, C16, C15, C16*E*, N4*E*). This type of distortion has been observed before (Setifi *et al.*, 2018 ▸, 2020 ▸).

**Figure 3:**
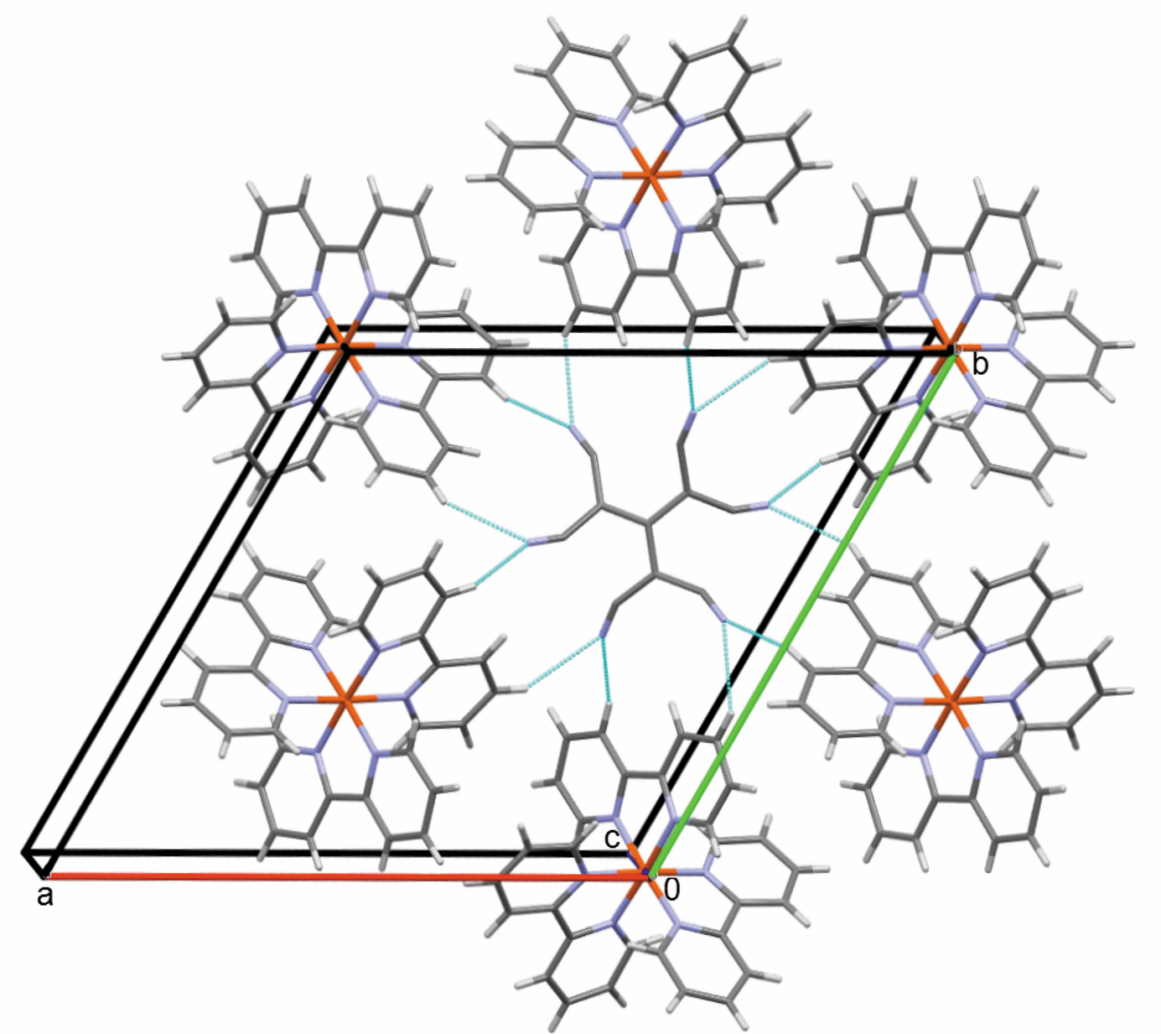
Partial packing diagram showing the hydrogen-bonding inter­actions C4—H4⋯N3 and C8—H8⋯N3 parallel to (001), as turquoise lines. Only one of the disordered set of sites is shown.

**Figure 4:**
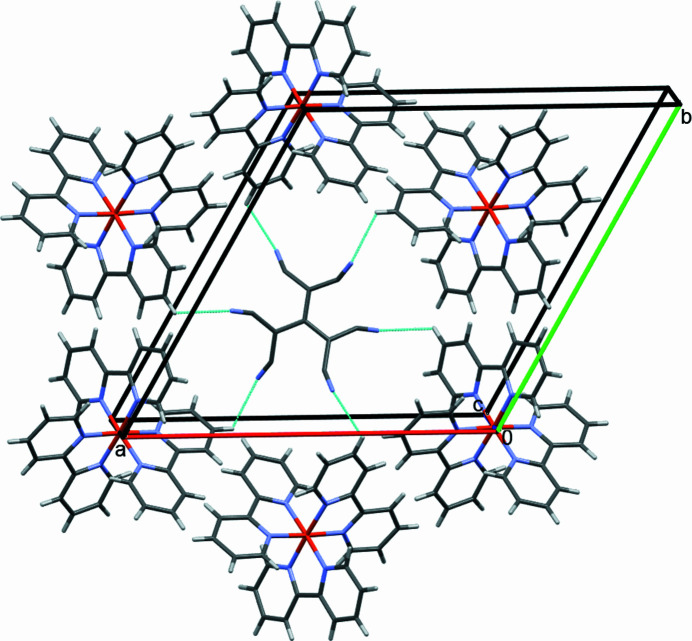
Partial packing diagram showing the hydrogen-bonding inter­actions C3—H3⋯N4 parallel to (001) as turquoise lines.. Only one of the disordered set of sites is shown.

**Figure 5:**
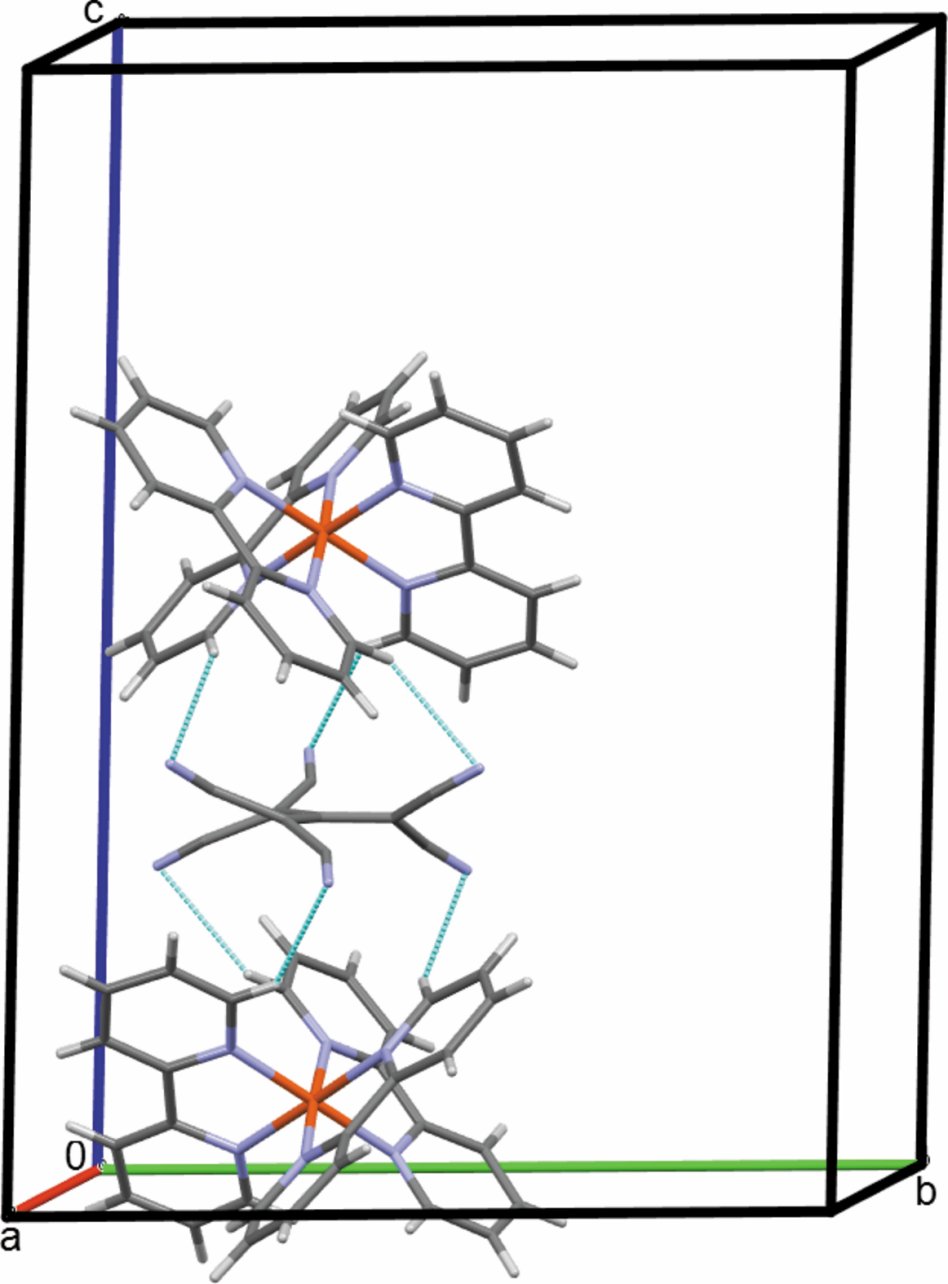
Partial packing diagram showing the hydrogen-bonding inter­actions C10—H10⋯N4 parallel to the crystallographic *c* axis, as turquoise lines. Only one of the disordered set of sites is shown.

Intra­molecular hydrogen bonds in the tris­(2,2′-bi­pyridine)­iron(II) cation are C1—H1⋯N1 and C10—H10⋯N2 between hydrogen atoms in *ortho* position and nitro­gen atoms from neighbouring di­pyridine units (Table 2 ▸). Further hydrogen bonds are present between cation and the anion consisting of C11—C12—C13—N3 with C4—H4⋯N3 and C8—H8⋯N3, which form complex layers parallel to (001) (Fig. 3 ▸). The other anion consisting of C14—C15—C16—N4 forms hydrogen bonds C3—H3⋯N4, forming layers that are also parallel to (001) (Fig. 4 ▸). Perpendicular to that are hydrogen bonds C10—H10⋯N4, which link the anion to two cations perpendicular to (001) (Fig. 5 ▸). All these inter­actions consolidate the crystal in a three-dimensional network of hydrogen bonds.

There are more than 100 crystal structures of tris­(2,2′-bi­pyridine)­iron(II) complexes listed in the Cambridge Structural Database (Groom *et al.*, 2016 ▸). From these are five crystal structures of closely related complexes containing the tris­(2,2′-bi­pyridine)­iron(II) cation and different polynitrile anions (Setifi, Setifi *et al.*, 2014 ▸; Potočňák *et al.*, 2014 ▸; Potočňák & Váhovská, 2014 ▸; Addala *et al.*, 2018 ▸).

## Synthesis and crystallization

A mixture of iron(II) bis­(tetra­fluoro­borate) hexa­hydrate (34 mg, 0.1 mmol), 2,2′-dipyridyl (16 mg, 0.1 mmol) and dipotassium tris­(di­cyano­methylid­ene)methane­diide (28 mg, 0.1 mmol), *N*,*N*-di­methyl­formamide (4 ml) and water (2 ml) was sonicated for 30 min. Then the reaction mixture was transferred to a Teflon-lined stainless steel reactor and placed in an oven. Subsequently, the temperature was kept 393 K for 3 days. After cooling to room temperature at a rate of 10 K h^−1^, red plate-shaped crystals of the title compound were obtained.

## Refinement

Crystal data, data collection and structure refinement details are summarized in Table 3 ▸. The two crystallographic independent tris­(di­cyano­methyl­idene)methane­diide ions are disordered over two atomic sites having equal occupancy.

## Supplementary Material

Crystal structure: contains datablock(s) I. DOI: 10.1107/S2414314625005036/bt4174sup1.cif

Structure factors: contains datablock(s) I. DOI: 10.1107/S2414314625005036/bt4174Isup2.hkl

CCDC reference: 2456126

Additional supporting information:  crystallographic information; 3D view; checkCIF report

## Figures and Tables

**Table 1:** Selected geometric parameters (Å, °)

Fe1—N1	1.9688 (13)	C13—N3	1.067 (2)
Fe1—N2	1.9734 (13)	C14—C15	1.421 (3)
C11—C12	1.422 (3)	C15—C16	1.482 (4)
C12—C13	1.473 (3)	C16—N4	1.079 (3)
			
N1^i^—Fe1—N1	96.73 (5)	N1—Fe1—N2	81.40 (5)
N1^i^—Fe1—N2	85.75 (5)	N2—Fe1—N2^i^	96.18 (5)
N1^ii^—Fe1—N2	177.07 (5)		

Symmetry codes: (i) 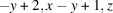; (ii) 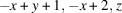.

**Table 2:** Hydrogen-bond geometry (Å, °)

*D*—H⋯*A*	*D*—H	H⋯*A*	*D*⋯*A*	*D*—H⋯*A*
C1—H1⋯N1^ii^	0.93	2.61	3.121 (2)	115
C10—H10⋯N2^i^	0.93	2.60	3.116 (2)	115
C4—H4⋯N3^iii^	0.93	2.45	3.333 (3)	159
C8—H8⋯N3^iv^	0.93	2.68	3.476 (3)	144
C3—H3⋯N4^iii^	0.93	2.65	3.244 (3)	122
C10—H10⋯N4^v^	0.93	2.68	3.437 (3)	139

Symmetry codes: (i) 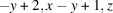; (ii) 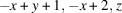; (iii) 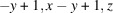; (iv) 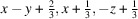; (v) 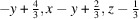.

**Table 3:** Experimental details

Crystal data	
Chemical formula	[Fe(C_10_H_8_N_2_)_3_](C_10_N_6_)
*M*_r_	728.56
Crystal system, space group	Trigonal, *R* 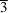 :*H*
Temperature (K)	298
*a*, *c* (Å)	17.0276 (3), 21.6388 (5)
*V* (Å^3^)	5433.4 (2)
*Z*	6
Radiation type	Mo *K*α
μ (mm^−1^)	0.46
Crystal size (mm)	0.45 × 0.28 × 0.15

Data collection	
Diffractometer	Bruker D8 VENTURE Duo
Absorption correction	Multi-scan (*SADABS*; Krause *et al.*, 2015 ▸)
*T*_min_, *T*_max_	0.875, 0.922
No. of measured, independent and observed [*I* > 2σ(*I*)] reflections	32654, 2780, 2269
*R*_int_	0.045
(sin θ/λ)_max_ (Å^−1^)	0.649

Refinement	
*R*[*F*^2^ > 2σ(*F*^2^)], *wR*(*F*^2^), *S*	0.033, 0.091, 1.05
No. of reflections	2780
No. of parameters	170
H-atom treatment	H-atom parameters constrained
Δρ_max_, Δρ_min_ (e Å^−3^)	0.22, −0.35

Computer programs: *APEX4* and *SAINT* (Bruker, 2021 ▸), *SHELXT* (Sheldrick, 2015*a* ▸), *SHELXL2019/2* (Sheldrick, 2015*b* ▸) and *ORTEP-3 for Windows* (Farrugia, 2012 ▸).

## full crystallographic data

### Tris(2,2'-bipyridine)iron(II) tris(dicyanomethylidene)methanediide Crystal data

**Table:**

[Fe(C_10_H_8_N_2_)_3_](C_10_N_6_)	*D*_x_ = 1.336 Mg m^−^^3^
*M**_r_* = 728.56	Mo *K*α radiation, λ = 0.71073 Å
Trigonal, *R*3:*H*	Cell parameters from 5342 reflections
*a* = 17.0276 (3) Å	θ = 2.8–28.4°
*c* = 21.6388 (5) Å	µ = 0.46 mm^−^^1^
*V* = 5433.4 (2) Å^3^	*T* = 298 K
*Z* = 6	Plate, red
*F*(000) = 2244	0.45 × 0.28 × 0.15 mm

### Tris(2,2'-bipyridine)iron(II) tris(dicyanomethylidene)methanediide Data collection

**Table:**

Bruker D8 VENTURE Duo diffractometer	2780 independent reflections
Radiation source: sealed tube	2269 reflections with *I* > 2σ(*I*)
TRIUMPH graphite monochromator	*R*_int_ = 0.045
ω and φ scans	θ_max_ = 27.5°, θ_min_ = 2.3°
Absorption correction: multi-scan (SADABS; Krause *et al.*, 2015)	*h* = −22→22
*T*_min_ = 0.875, *T*_max_ = 0.922	*k* = −22→22
32654 measured reflections	*l* = −28→27

### Tris(2,2'-bipyridine)iron(II) tris(dicyanomethylidene)methanediide Refinement

**Table:**

Refinement on *F*^2^	Primary atom site location: structure-invariant direct methods
Least-squares matrix: full	Hydrogen site location: inferred from neighbouring sites
*R*[*F*^2^ > 2σ(*F*^2^)] = 0.033	H-atom parameters constrained
*wR*(*F*^2^) = 0.091	*w* = 1/[σ^2^(*F*_o_^2^) + (0.0367*P*)^2^ + 5.2576*P*] where *P* = (*F*_o_^2^ + 2*F*_c_^2^)/3
*S* = 1.05	(Δ/σ)_max_ < 0.001
2780 reflections	Δρ_max_ = 0.22 e Å^−^^3^
170 parameters	Δρ_min_ = −0.35 e Å^−^^3^
0 restraints	

### Tris(2,2'-bipyridine)iron(II) tris(dicyanomethylidene)methanediide Special details

**Table:**

Geometry. All esds (except the esd in the dihedral angle between two l.s. planes) are estimated using the full covariance matrix. The cell esds are taken into account individually in the estimation of esds in distances, angles and torsion angles; correlations between esds in cell parameters are only used when they are defined by crystal symmetry. An approximate (isotropic) treatment of cell esds is used for estimating esds involving l.s. planes.
Refinement. All non-hydrogen atoms were refined anisotropically. Hydrogen atoms were placed in idealized positions and refined with a riding model with C–H = 0.93 Å and with *U*_iso_(H) = 1.2 *U*_eq_(C).

### Tris(2,2'-bipyridine)iron(II) tris(dicyanomethylidene)methanediide Fractional atomic coordinates and isotropic or equivalent isotropic displacement parameters (Å^2^)

**Table:**

	*x*	*y*	*z*	*U*_iso_*/*U*_eq_	Occ. (<1)
Fe1	1.000000	1.000000	0.24788 (2)	0.03504 (12)	
N1	0.89912 (9)	0.99778 (9)	0.29384 (6)	0.0386 (3)	
N2	0.89822 (9)	0.90276 (9)	0.20124 (5)	0.0391 (3)	
C1	0.90642 (12)	1.05483 (12)	0.33947 (7)	0.0475 (4)	
H1	0.963517	1.093679	0.355820	0.057*	
C2	0.83306 (13)	1.05808 (13)	0.36290 (8)	0.0562 (5)	
H2	0.840747	1.098700	0.394234	0.067*	
C3	0.74830 (13)	1.00060 (14)	0.33947 (9)	0.0590 (5)	
H3	0.697874	1.002039	0.354509	0.071*	
C4	0.73902 (12)	0.94059 (13)	0.29322 (9)	0.0536 (4)	
H4	0.682084	0.900635	0.277093	0.064*	
C5	0.81535 (11)	0.94053 (11)	0.27110 (7)	0.0408 (3)	
C6	0.81457 (11)	0.88191 (11)	0.22107 (7)	0.0423 (3)	
C7	0.73688 (13)	0.81059 (13)	0.19582 (9)	0.0593 (5)	
H7	0.679985	0.798009	0.209532	0.071*	
C8	0.74465 (14)	0.75829 (14)	0.15005 (10)	0.0685 (6)	
H8	0.693155	0.709862	0.132822	0.082*	
C9	0.82930 (14)	0.77881 (13)	0.13043 (9)	0.0609 (5)	
H9	0.836022	0.744194	0.099887	0.073*	
C10	0.90415 (12)	0.85113 (12)	0.15642 (7)	0.0491 (4)	
H10	0.961322	0.865004	0.142456	0.059*	
C11	0.333333	0.666667	0.166667	0.0315 (7)	
C12	0.27300 (19)	0.57138 (18)	0.16798 (14)	0.0356 (6)	0.5
C13	0.30840 (13)	0.51355 (11)	0.19074 (8)	0.0512 (4)	
N3	0.29929 (17)	0.45296 (12)	0.21194 (10)	0.0919 (7)	
C14	0.666667	0.333333	0.333333	0.0427 (8)	
C15	0.5716 (2)	0.2721 (3)	0.33372 (16)	0.0504 (8)	0.5
C16	0.51371 (14)	0.30692 (18)	0.35854 (9)	0.0707 (6)	
N4	0.45347 (15)	0.2971 (2)	0.38184 (11)	0.1096 (9)	

### Tris(2,2'-bipyridine)iron(II) tris(dicyanomethylidene)methanediide Atomic displacement parameters (Å^2^)

**Table:**

	*U*^11^	*U*^22^	*U*^33^	*U*^12^	*U*^13^	*U*^23^
Fe1	0.03581 (14)	0.03581 (14)	0.03350 (19)	0.01790 (7)	0.000	0.000
N1	0.0407 (7)	0.0398 (7)	0.0371 (6)	0.0215 (6)	−0.0010 (5)	−0.0020 (5)
N2	0.0416 (7)	0.0391 (7)	0.0361 (6)	0.0199 (6)	0.0000 (5)	−0.0012 (5)
C1	0.0495 (9)	0.0523 (10)	0.0438 (8)	0.0277 (8)	−0.0018 (7)	−0.0088 (7)
C2	0.0636 (11)	0.0616 (11)	0.0539 (10)	0.0391 (10)	0.0022 (9)	−0.0113 (8)
C3	0.0543 (11)	0.0693 (12)	0.0665 (11)	0.0408 (10)	0.0069 (9)	−0.0051 (9)
C4	0.0417 (9)	0.0602 (11)	0.0618 (10)	0.0277 (8)	−0.0010 (8)	−0.0054 (8)
C5	0.0399 (8)	0.0422 (8)	0.0423 (8)	0.0220 (7)	−0.0005 (6)	−0.0001 (6)
C6	0.0404 (8)	0.0414 (8)	0.0440 (8)	0.0196 (7)	−0.0028 (6)	−0.0026 (6)
C7	0.0424 (9)	0.0580 (11)	0.0672 (11)	0.0174 (9)	−0.0047 (8)	−0.0150 (9)
C8	0.0539 (11)	0.0600 (12)	0.0746 (13)	0.0158 (10)	−0.0121 (10)	−0.0258 (10)
C9	0.0646 (12)	0.0551 (11)	0.0564 (10)	0.0251 (9)	−0.0038 (9)	−0.0197 (9)
C10	0.0497 (9)	0.0508 (10)	0.0441 (8)	0.0231 (8)	0.0011 (7)	−0.0086 (7)
C11	0.0298 (10)	0.0298 (10)	0.0348 (16)	0.0149 (5)	0.000	0.000
C12	0.0305 (13)	0.0306 (14)	0.0452 (15)	0.0148 (11)	−0.0008 (12)	−0.0012 (11)
C13	0.0638 (11)	0.0358 (8)	0.0563 (10)	0.0267 (8)	−0.0048 (8)	0.0013 (7)
N3	0.133 (2)	0.0465 (10)	0.0953 (14)	0.0447 (12)	−0.0088 (14)	0.0143 (10)
C14	0.0450 (13)	0.0450 (13)	0.0380 (18)	0.0225 (6)	0.000	0.000
C15	0.0471 (19)	0.052 (2)	0.0479 (17)	0.0216 (16)	0.0006 (14)	0.0005 (15)
C16	0.0480 (11)	0.1100 (18)	0.0564 (11)	0.0412 (12)	−0.0070 (9)	−0.0067 (11)
N4	0.0566 (12)	0.183 (3)	0.0910 (15)	0.0611 (15)	−0.0082 (11)	−0.0235 (16)

### Tris(2,2'-bipyridine)iron(II) tris(dicyanomethylidene)methanediide Geometric parameters (Å, º)

**Table:**

Fe1—N1^i^	1.9688 (13)	C9—H9	0.9300
Fe1—N1^ii^	1.9688 (13)	C10—H10	0.9300
Fe1—N1	1.9688 (13)	C11—C12^iii^	1.422 (3)
Fe1—N2	1.9734 (13)	C11—C12^iv^	1.422 (3)
Fe1—N2^i^	1.9734 (13)	C11—C12	1.422 (3)
Fe1—N2^ii^	1.9734 (13)	C11—C12^v^	1.422 (3)
N1—C1	1.346 (2)	C11—C12^vi^	1.422 (3)
N1—C5	1.355 (2)	C11—C12^vii^	1.422 (3)
N2—C10	1.346 (2)	C12—C12^iii^	1.423 (3)
N2—C6	1.354 (2)	C12—C12^vii^	1.423 (3)
C1—C2	1.375 (2)	C12—C13	1.473 (3)
C1—H1	0.9300	C12—C13^vii^	1.492 (3)
C2—C3	1.373 (3)	C13—N3	1.067 (2)
C2—H2	0.9300	C14—C15^viii^	1.421 (3)
C3—C4	1.382 (3)	C14—C15^ix^	1.421 (3)
C3—H3	0.9300	C14—C15	1.421 (3)
C4—C5	1.386 (2)	C14—C15^x^	1.421 (3)
C4—H4	0.9300	C14—C15^xi^	1.421 (3)
C5—C6	1.468 (2)	C14—C15^xii^	1.422 (3)
C6—C7	1.384 (2)	C15—C15^viii^	1.421 (3)
C7—C8	1.382 (3)	C15—C15^xii^	1.422 (3)
C7—H7	0.9300	C15—C16	1.482 (4)
C8—C9	1.370 (3)	C15—C16^xii^	1.482 (4)
C8—H8	0.9300	C16—N4	1.079 (3)
C9—C10	1.374 (2)		
			
N1^i^—Fe1—N1^ii^	96.74 (5)	C12^iii^—C11—C12^v^	60.040 (9)
N1^i^—Fe1—N1	96.73 (5)	C12^iv^—C11—C12^v^	119.960 (9)
N1^ii^—Fe1—N1	96.74 (5)	C12—C11—C12^v^	119.960 (9)
N1^i^—Fe1—N2	85.75 (5)	C12^iii^—C11—C12^vi^	119.964 (10)
N1^ii^—Fe1—N2	177.07 (5)	C12^iv^—C11—C12^vi^	60.037 (9)
N1—Fe1—N2	81.40 (5)	C12—C11—C12^vi^	180.0
N1^i^—Fe1—N2^i^	81.40 (5)	C12^v^—C11—C12^vi^	60.043 (9)
N1^ii^—Fe1—N2^i^	85.75 (5)	C12^iii^—C11—C12^vii^	119.962 (9)
N1—Fe1—N2^i^	177.07 (5)	C12^iv^—C11—C12^vii^	60.037 (9)
N2—Fe1—N2^i^	96.18 (5)	C12—C11—C12^vii^	60.042 (9)
N1^i^—Fe1—N2^ii^	177.07 (5)	C12^v^—C11—C12^vii^	180.0
N1^ii^—Fe1—N2^ii^	81.40 (5)	C12^vi^—C11—C12^vii^	119.955 (9)
N1—Fe1—N2^ii^	85.75 (5)	C11—C12—C12^iii^	59.978 (4)
N2—Fe1—N2^ii^	96.18 (5)	C11—C12—C12^vii^	59.981 (5)
N2^i^—Fe1—N2^ii^	96.18 (5)	C12^iii^—C12—C12^vii^	119.84 (3)
C1—N1—C5	118.01 (14)	C11—C12—C13	117.8 (2)
C1—N1—Fe1	126.27 (11)	C12^iii^—C12—C13	62.0 (2)
C5—N1—Fe1	115.13 (10)	C12^vii^—C12—C13	162.7 (4)
C10—N2—C6	118.00 (14)	C11—C12—C13^vii^	116.5 (2)
C10—N2—Fe1	126.53 (11)	C12^iii^—C12—C13^vii^	156.1 (4)
C6—N2—Fe1	115.18 (10)	C12^vii^—C12—C13^vii^	60.6 (2)
N1—C1—C2	122.79 (16)	C13—C12—C13^vii^	125.6 (2)
N1—C1—H1	118.6	N3—C13—C12	151.6 (3)
C2—C1—H1	118.6	N3—C13—C12^iii^	150.9 (2)
C3—C2—C1	119.18 (16)	C12—C13—C12^iii^	57.34 (18)
C3—C2—H2	120.4	C15^viii^—C14—C15^ix^	180.0 (2)
C1—C2—H2	120.4	C15^viii^—C14—C15	60.003 (4)
C2—C3—C4	119.04 (17)	C15^ix^—C14—C15	119.996 (4)
C2—C3—H3	120.5	C15^viii^—C14—C15^x^	60.004 (3)
C4—C3—H3	120.5	C15^ix^—C14—C15^x^	119.997 (5)
C3—C4—C5	119.36 (17)	C15—C14—C15^x^	119.997 (3)
C3—C4—H4	120.3	C15^viii^—C14—C15^xi^	120.000 (4)
C5—C4—H4	120.3	C15^ix^—C14—C15^xi^	60.001 (3)
N1—C5—C4	121.62 (15)	C15—C14—C15^xi^	180.0
N1—C5—C6	113.92 (13)	C15^x^—C14—C15^xi^	60.006 (4)
C4—C5—C6	124.45 (15)	C15^viii^—C14—C15^xii^	119.998 (4)
N2—C6—C7	121.57 (15)	C15^ix^—C14—C15^xii^	60.001 (4)
N2—C6—C5	113.87 (13)	C15—C14—C15^xii^	60.005 (4)
C7—C6—C5	124.56 (15)	C15^x^—C14—C15^xii^	180.0
C8—C7—C6	119.39 (18)	C15^xi^—C14—C15^xii^	119.991 (3)
C8—C7—H7	120.3	C14—C15—C15^viii^	59.996 (2)
C6—C7—H7	120.3	C14—C15—C15^xii^	59.999 (2)
C9—C8—C7	119.04 (17)	C15^viii^—C15—C15^xii^	119.984 (14)
C9—C8—H8	120.5	C14—C15—C16	116.8 (3)
C7—C8—H8	120.5	C15^viii^—C15—C16	61.4 (3)
C8—C9—C10	119.21 (17)	C15^xii^—C15—C16	159.4 (4)
C8—C9—H9	120.4	C14—C15—C16^xii^	116.7 (3)
C10—C9—H9	120.4	C15^viii^—C15—C16^xii^	157.4 (4)
N2—C10—C9	122.77 (16)	C15^xii^—C15—C16^xii^	61.3 (3)
N2—C10—H10	118.6	C16—C15—C16^xii^	126.5 (3)
C9—C10—H10	118.6	N4—C16—C15	151.5 (3)
C12^iii^—C11—C12^iv^	180.00 (17)	N4—C16—C15^viii^	150.8 (3)
C12^iii^—C11—C12	60.039 (9)	C15—C16—C15^viii^	57.3 (2)
C12^iv^—C11—C12	119.960 (9)		
			
C5—N1—C1—C2	−1.0 (2)	C12^v^—C11—C12—C13	19.6 (4)
Fe1—N1—C1—C2	169.70 (14)	C12^vii^—C11—C12—C13	−160.4 (4)
N1—C1—C2—C3	0.5 (3)	C12^iii^—C11—C12—C13^vii^	−153.1 (4)
C1—C2—C3—C4	0.4 (3)	C12^iv^—C11—C12—C13^vii^	26.9 (4)
C2—C3—C4—C5	−0.8 (3)	C12^v^—C11—C12—C13^vii^	−157.06 (12)
C1—N1—C5—C4	0.7 (2)	C12^vii^—C11—C12—C13^vii^	22.94 (12)
Fe1—N1—C5—C4	−171.07 (13)	C11—C12—C13—N3	153.0 (4)
C1—N1—C5—C6	179.30 (14)	C12^iii^—C12—C13—N3	176.1 (5)
Fe1—N1—C5—C6	7.55 (17)	C12^vii^—C12—C13—N3	75.2 (8)
C3—C4—C5—N1	0.2 (3)	C13^vii^—C12—C13—N3	−30.7 (6)
C3—C4—C5—C6	−178.27 (17)	C11—C12—C13—C12^iii^	−23.06 (8)
C10—N2—C6—C7	−0.9 (2)	C12^vii^—C12—C13—C12^iii^	−100.9 (7)
Fe1—N2—C6—C7	−175.08 (14)	C13^vii^—C12—C13—C12^iii^	153.2 (4)
C10—N2—C6—C5	178.43 (14)	C15^ix^—C14—C15—C15^viii^	180.0
Fe1—N2—C6—C5	4.22 (17)	C15^x^—C14—C15—C15^viii^	1.2 (5)
N1—C5—C6—N2	−7.7 (2)	C15^xii^—C14—C15—C15^viii^	−178.8 (5)
C4—C5—C6—N2	170.89 (15)	C15^viii^—C14—C15—C15^xii^	178.8 (5)
N1—C5—C6—C7	171.59 (16)	C15^ix^—C14—C15—C15^xii^	−1.2 (5)
C4—C5—C6—C7	−9.8 (3)	C15^x^—C14—C15—C15^xii^	180.0
N2—C6—C7—C8	1.2 (3)	C15^viii^—C14—C15—C16	−24.35 (14)
C5—C6—C7—C8	−178.05 (18)	C15^ix^—C14—C15—C16	155.65 (14)
C6—C7—C8—C9	−0.5 (3)	C15^x^—C14—C15—C16	−23.2 (5)
C7—C8—C9—C10	−0.4 (3)	C15^xii^—C14—C15—C16	156.8 (5)
C6—N2—C10—C9	−0.1 (3)	C15^viii^—C14—C15—C16^xii^	154.5 (5)
Fe1—N2—C10—C9	173.37 (14)	C15^ix^—C14—C15—C16^xii^	−25.5 (5)
C8—C9—C10—N2	0.8 (3)	C15^x^—C14—C15—C16^xii^	155.66 (14)
C12^iv^—C11—C12—C12^iii^	180.002 (1)	C15^xii^—C14—C15—C16^xii^	−24.34 (14)
C12^v^—C11—C12—C12^iii^	−4.0 (4)	C14—C15—C16—N4	−149.5 (4)
C12^vii^—C11—C12—C12^iii^	176.0 (4)	C15^viii^—C15—C16—N4	−173.6 (5)
C12^iii^—C11—C12—C12^vii^	−176.0 (4)	C15^xii^—C15—C16—N4	−74.0 (9)
C12^iv^—C11—C12—C12^vii^	4.0 (4)	C16^xii^—C15—C16—N4	31.7 (7)
C12^v^—C11—C12—C12^vii^	180.0	C14—C15—C16—C15^viii^	24.01 (10)
C12^iii^—C11—C12—C13	23.55 (11)	C15^xii^—C15—C16—C15^viii^	99.5 (7)
C12^iv^—C11—C12—C13	−156.45 (11)	C16^xii^—C15—C16—C15^viii^	−154.7 (5)

Symmetry codes: (i) −*y*+2, *x*−*y*+1, *z*; (ii) −*x*+*y*+1, −*x*+2, *z*; (iii) *x*−*y*+2/3, *x*+1/3, −*z*+1/3; (iv) −*x*+*y*, −*x*+1, *z*; (v) −*y*+1, *x*−*y*+1, *z*; (vi) −*x*+2/3, −*y*+4/3, −*z*+1/3; (vii) *y*−1/3, −*x*+*y*+1/3, −*z*+1/3; (viii) *y*+1/3, −*x*+*y*+2/3, −*z*+2/3; (ix) −*y*+1, *x*−*y*, *z*; (x) −*x*+*y*+1, −*x*+1, *z*; (xi) −*x*+4/3, −*y*+2/3, −*z*+2/3; (xii) *x*−*y*+1/3, *x*−1/3, −*z*+2/3.

### Tris(2,2'-bipyridine)iron(II) tris(dicyanomethylidene)methanediide Hydrogen-bond geometry (Å, º)

**Table:**

*D*—H···*A*	*D*—H	H···*A*	*D*···*A*	*D*—H···*A*
C1—H1···N1^ii^	0.93	2.61	3.121 (2)	115
C10—H10···N2^i^	0.93	2.60	3.116 (2)	115
C4—H4···N3^v^	0.93	2.45	3.333 (3)	159
C8—H8···N3^iii^	0.93	2.68	3.476 (3)	144
C3—H3···N4^v^	0.93	2.65	3.244 (3)	122
C10—H10···N4^xiii^	0.93	2.68	3.437 (3)	139

Symmetry codes: (i) −*y*+2, *x*−*y*+1, *z*; (ii) −*x*+*y*+1, −*x*+2, *z*; (iii) *x*−*y*+2/3, *x*+1/3, −*z*+1/3; (v) −*y*+1, *x*−*y*+1, *z*; (xiii) −*y*+4/3, *x*−*y*+2/3, *z*−1/3.
